# Evaluation of Ocular Biometric Parameters Following Cataract Surgery

**DOI:** 10.3390/reports6010003

**Published:** 2023-01-18

**Authors:** Cosmin Adrian Teodoru, Maria-Emilia Cerghedean-Florea, Ciprian Tănăsescu, Horațiu Dura, Radu Fleacă, Mihnea Munteanu, Horia Stanca, Florina Georgeta Popescu, Mihai Dan Roman, Gheorghe Zsolt Nicula, Horea Vladi Matei, Mihaela Laura Vică

**Affiliations:** 1 Clinical Surgical Department, Faculty of Medicine, “Lucian Blaga” University Sibiu, 550169 Sibiu, Romania; 2 Third Clinico-Surgical Department, Faculty of Medicine, “Lucian Blaga“ University, 550169 Sibiu, Romania; 3 Preclinical Department, Faculty of Medicine, “Lucian Blaga“ University, 550169 Sibiu, Romania; 4 Surgical Clinical Department, Faculty of General Medicine ‘’Lucian Blaga’’ University of Sibiu, 550169 Sibiu, Romania; 5 Department of Ophthalmology, “Victor Babes” University of Medicine and Pharmacy, 300041 Timisoara, Romania; 6 Department of Ophthalmology, “Carol Davila” University of Medicine and Pharmacy, 020021 Bucharest, Romania; 7 Department of Occupational Medicine, University of Medicine and Pharmacy Victor Babes, 300041 Timisoara, Romania; 8 Department of Orthopedics and Trauma, Faculty of Medicine, “Lucian Blaga” University, 550169 Sibiu, Romania; 9 Department of Cellular and Molecular Biology, “Iuliu Haţieganu” University of Medicine and Pharmacy, 400012 Cluj-Napoca, Romania; 10 Institute of Legal Medicine, 400006 Cluj-Napoca, Romania

**Keywords:** cataract, phacoemulsification, ocular biometric parameters

## Abstract

Background: The aim of this study was to highlight the structural changes in patients with cataract following surgery and the repercussions on the anterior pole. Methods: A total of 83 patients diagnosed with cataract who underwent uneventful phacoemulsification was included. Every patient was examined one week prior to and two weeks after the surgery. Pre- and postoperative assessment included examination of the anterior and posterior segment, keratometry, and optical biometry. Results: The pre- vs. postoperative axial length (AL) mean difference was 0.07 ± 0.18 mm (*p* < 0.001).The mean difference of the postoperative anterior chamber depth (ACD) vs. preoperative ACD values (1.11 ± 0.50 mm) was also statistically significant (*p* < 0.001). The linear regression function postoperative central corneal thickness (CCT) = 0.9004 × (preoperative CCT) + 0.0668, where it characterized a reduced positive correlation (R^2^) of 68.89% between the postoperative CCT and preoperative CCT. The mean pre-/post-operative differences in the K1 values were 0.08 ± 0.38 D, with a statistically significant difference between the two datasets (*p* = 0.0152). The mean pre/postoperative difference in the K2 values was 0.002 ± 0.58 D (*p* = 0.4854). Conclusions: ACD deepened significantly postoperatively. Regarding AL, there was a decrease after surgery, and a very good positive correlation between the post and preoperative values. The CCT values decreased with age. The 2.2-mm corneal incision during cataract surgery resulted in a relatively small postoperative residual astigmatism.

## 1. Introduction

Cataracts are the leading cause of vision loss worldwide [1]. An estimated 95 million people worldwide are affected by cataracts [2]. The overall prevalence of cataracts in adults over the age of 50 has been estimated to be 47.8% [3]. Age is a determining factor in ocular degeneration at the anterior and posterior pole [4]. Additionally, other eye and general diseases can have a significant impact on the eye structures and ocular surgery, thus influencing the visual prognosis of the patients [5,6,7,8]. 

Cataract is age-related, increasing from 5% for those aged 52–62 to 30% for the 60–69 year old group and 64% for the population over 70 years [9,10]. Age-related cataracts are by far the most common variety occurring, often asymmetrically, in both men and women over the age of 60 [11]. A significant hereditary tendency manifesting at an increasingly younger age in successive generations has been observed [12]. 

The volume of worldwide cataract surgery, the only treatment option at present, has gradually increased in tandem with the life expectancy of the general population to become the most common ophthalmic surgery in many countries. Phacoemulsification is the gold standard for cataract surgery in developed countries [13]. It implies a smaller corneal incision, shorter healing time, reduced postoperative astigmatism, and faster visual recovery [14]. A corneal relaxation incision can correct small degrees of astigmatism, but for preoperative corneal astigmatism ≥1.0 diopters, the implantation of a toric intraocular lens should be considered [15,16].

Accurate biometric measurements are an essential tool in clinical ophthalmic practice, especially when performing surgical interventions on the anterior segment. These are fundamental for accurate intra-ocular lens (IOL) power calculation based on formulas derived from normal ocular biometric parameters.

The technology used by Topcon Aladdin (Topcon Corporation, Tokyo, Japan) is based on optical low-coherence interferometry (OLCI) using an 830 nm superluminescent diode [17]. Having a good penetrability on dense cataracts, this optical biometrics and corneal topography device, launched in 2012, is able to simultaneously perform a series of anatomical measurements of the anterior segment. It can also be used in cases of pseudophakic, aphakia, or silicone oil [18]. The results generated by the Topcon Aladdin device are similar to those obtained using other types of technology employed for optical biometrics [17,19,20,21]. However, few studies have evaluated the reliability of the Topcon Aladdin device regarding the pre- and postoperative parameters obtained for patients with pseudophakic [18].

Regarding the biometric variables, it is known that following cataract surgery, these parameters of the anterior corneal segment are exposed to a number of changes that may have a significant impact on the visual function of the patients [22,23,24,25]. 

The aim of this paper was to highlight the ocular structural changes occurring in patients with age-related cataracts following cataract surgery and their repercussions on the anterior pole, to assess pre- and postoperative values of the ocular biometric parameters such as the anterior chamber depth (ACD), axial length (AL), central corneal thickness (CCT), lens thickness (LT), or variations in the degree of anterior corneal astigmatism induced by the keratometric parameters K1 and K2, and to investigate the interdependence of these parameters.

## 2. Materials and Methods

This prospective clinical study was conducted over a period of two months (July–September 2022) on a group of 83 patients (103 eyes) diagnosed with cataract at Arcada Clinic, Sibiu County, Romania, who were subjected to cataract surgery by phacoemulsification at the same clinic, 20 of them in both eyes. Informed consent was obtained from all subjects involved in the study. The study was conducted in accordance with the Declaration of Helsinki and by the ethics committee of the “Lucian Blaga” University in Sibiu (No. 9, date of approval: 29 July 2022). 

**Figure 1:**
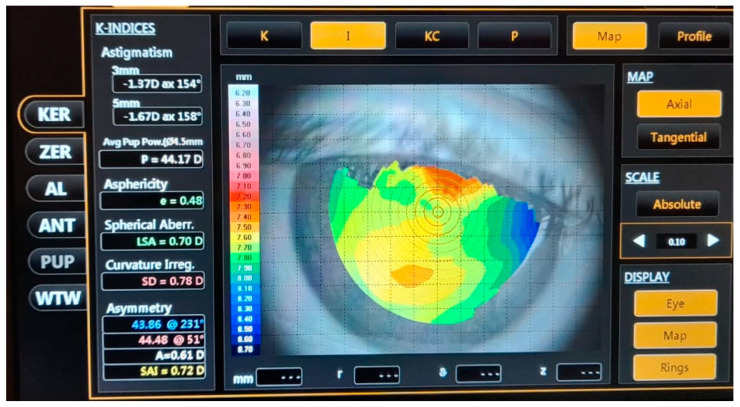
Map of the postoperative astigmatism measured using the Topcon Aladdin Optical Biometer and Corneal Topographer (Topcon Corporation, Tokyo, Japan).

The selection process was based on the following inclusion criteria: patients with cataract, who underwent phacoemulsification surgery combined with intraocular lens implantation, presented no glaucoma, corneal pathology, or intraoperative and postoperative complications following surgery, and agreed to have their biometric parameters reassessed two weeks after the intervention. Patients who suffered intraoperative complications, trauma, or postoperative infections, or those whose surgical procedure required any deviation from the standard duration (20–25 min) or operating steps were excluded from the study. Every patient was examined one week prior to and two weeks after the surgical intervention. Pre- and postoperative assessment included visual acuity checking, intraocular pressure measurement, examination of the fundus, anterior segment and appendix, keratometry, and optical biometry. Seven biometric variables were simultaneously measured by OLCI using a Topcon Aladdin device (Figure 1): AL, ACD, CCT, and LT, K1, K2, and the degree of anterior corneal astigmatism (CYL), pre- and postoperative corneal power values (mm) were converted into diopters using the formulae K (D) = 337.5/K (mm) based on the equipment’s keratometric refractive index of 1.337512. Measurements were conducted in triplicate and automatically averaged based on the biometric and keratometric data. All of these tests were performed after pupil dilation and were performed by the same examiner.

The surgical procedure consisted of several stages. All surgeries were performed by a single surgeon using the same machine (Infiniti^®^, OZil^®^ Torsional hand piece; Alcon Laboratories, Inc., Fort Worth, TX, USA). Ophthalmic drops in tropicamide solution and topical anesthesia were first administered for mydriasis, then a 2.2-mm corneal incision in the upper part of the cornea and two auxiliary lateral incisions were made. A viscoelastic material was used to protect the corneal endothelium and to keep the anterior chamber space in a relaxed state. A continuous circular capsulotomy was performed with the capsulotomy forceps. Hydrodissection and hydrodelination were then performed so that the nucleus could rotate freely, and nuclear fragmentation was performed using the divide and conquer and stop and chop techniques. The posterior chamber intraocular lens was implanted in the capsular bag following phacoemulsification. Different types of intraocular lenses were chosen according to the patients’ needs.

Data were statistically analyzed using the Microsoft Office Excel application (Microsoft Corp., Redmond, DC, USA). The t-paired test was used to compare the patients’ preoperative and postoperative results. Linear associations between the quantitative variables were determined based on the Pearson correlation coefficient. A *p* value below 0.05 was considered statistically significant.

## 3. Results

The 83 cataract patients aged 50 to 90 years (mean age 72.05 ± 7.26 years) were subjected to surgical interventions and had no intraoperative or postoperative complications. Of the total 103 interventions (53 on the right eye and 50 on the left eye), 64 (62.1%) involved female patients aged 64–88. The distribution by sex and age groups are presented in Table 1.

The centrality and dispersion indicators of the biometric and keratometric ocular parameters measured with the Topcon Aladdin Optical Biometer and Corneal Topographer one week before and two weeks after the surgical interventions are shown in Table 2.

**Figure 2:**
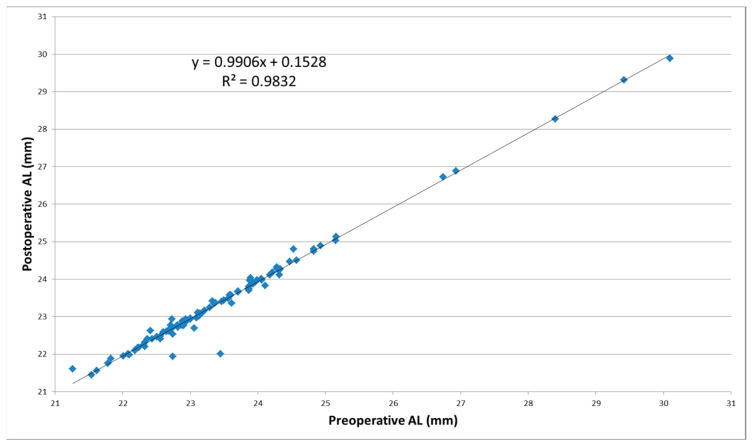
Correlation between the postoperative and preoperative AL values.

Most preoperative AL values were within the normal limits (22–26 mm), five each being below 22 (presbyopia) and over 26 mm (myopia). The pre- vs. postoperative AL mean difference was 0.07 ± 0.18 mm (*p* < 0.001), with a slight decrease after surgery. The linear regression function postoperative AL = 0.9906 preoperative AL +0.1528 characterizes a very good positive correlation of 98.32% between postoperative AL and preoperative AL (Figure 2).

The mean difference of postoperative ACD vs. preoperative ACD values (1.11 ± 0.50 mm) was also statistically significant (*p* < 0.001). The linear regression function postoperative ACD = 0.5241 preoperative ACD +2.6427 indicated a weak positive correlation of 27.46% between the postoperative ACD and preoperative ACD. 

The LT mean difference before and after cataract surgery (3.69 ± 0.36 mm) was also statistically significant (*p* < 0.001). The linear regression function postoperative LT = 0.0222 preoperative LT +0.6903 indicated a very weak positive correlation of 0.44% between the postoperative LT and pre-operative LT.

A significant weak negative correlation of 19.28% (*p* < 0.001) was found when applying the linear regression function for preoperative ACD (−0.6477) vs. preoperative LT (+6.1357). When modeling the relationship between the postoperative ACD (−0.1412) and preoperative LT (+4.9695), we found a very weak negative correlation of 0.92% (*p* < 0.05). Similarly, a very weak negative correlation of 0.37% (*p* < 0.01) was found for the postoperative ACD (−0.2687) vs. postoperative LT (+4.5484). 

More than half of the CCT values were within the normal limits (505–567 µm), both preoperatively (61 measurements, 59.2%) and postoperatively (59 measurements, 57.3%). Values above 567 µm were found in 32 preoperative measurements (31.1%) and 35 postoperative measurements (34%), while values below 505 µm were observed in 10 preoperative measurements (9.7%) and nine postoperative measurements (8.7%). The mean difference of postoperative vs. preoperative CCT values was 12.36 ± 21.61 µm, with a statistically significant increase after cataract surgery (*p* < 0.01). We found slightly decreased mean CCT values in the 60s group compared to the 50s and significantly decreased in the 80s compared to the 70s. Preoperative means were 552 ± 19.36 µm for the 50–59 year old age group, 548 ± 33.61 µm for the 60–69 year old group, 551 ± 36.55 µm for the 70–79 year old group, and 522 ± 32.23 µm for the 80–90 year old group. Similarly, the postoperative CCT means were 569 ± 21.92 (50–59 years), 553 ± 35.88 µm (60–69 years), 567 ± 39.69 µm (70–79 years), and 541 ± 37.69 µm (80–90 years)

**Figure 3:**
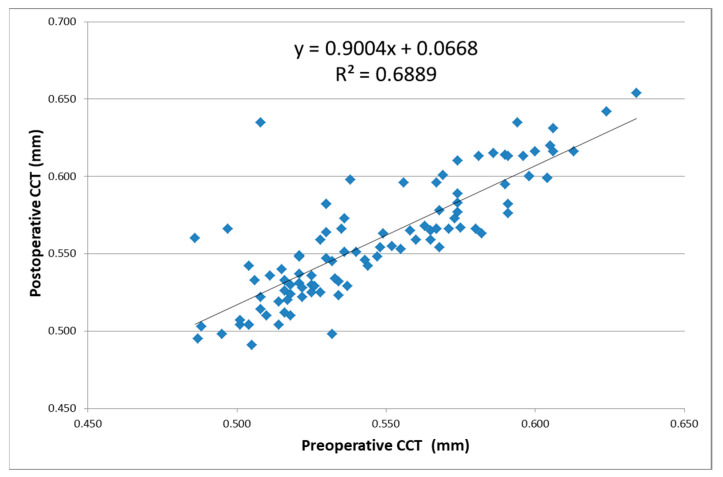
Correlation between the postoperative and preoperative central corneal thickness (CCT) values.

The linear regression function postoperative central corneal thickness (CCT) = 0.9004 × (preoperative CCT) + 0.0668, where it characterizes a reduced positive correlation (R^2^) of 68.89% between the postoperative CCT and preoperative CCT, as seen in Figure 3.

When assessing the degree of preoperative corneal astigmatism, 38 CYL values were found to be within the (0; −0.5) interval, 36 within the (−0.5; −1) interval, 17 within the (−1; −1.5) interval, four within the (−1.5; −2) interval, and three below −2 CYL, while in five cases, values between 0 and 1 CYL were observed. For all patients, the quartile values were −0.96 for Q1, −0.59 for Q2 (median), and −0.30 for Q3.

Regarding the postoperative evaluation, figures were 26 (25,2%) for the (0; −0.5) CYL interval, 47 (45,6%) for the (−0.5; −1) CYL interval, 17 within the (−1; −1.5) interval, six within the (−1.5; −2) interval, three below −2 CYL, and four between 0 and 1 CYL, respectively. The quartile values at the postoperative assessment were −1.02 for Q1, −0.66 for Q2 (median), and −0.42 for Q3.

Preoperative values varied between −3.65 and 0.97 CYL (mean −0.76 ± 0.69, median −0.64) for female patients, while for males, the CYL values were found within the (−2.19; 0) interval (mean −0.61 ± 0.44, median −0.53). Postoperative assessment revealed an average degree of anterior corneal astigmatism of −0.89 ± 0.63 CYL (values between −3.55 and 0, median −0.72) in female patients, and of −0.64 ± 0.39 (median −0.54) CYL in males (values between −1.79 and 0, median −0.54). The mean postoperative vs. preoperative differences in the degree of anterior corneal astigmatism were −0.09 ± 0.47 CYL (median −0.06), with a statistically significant decrease after cataract surgery (*p* = 0.0296). The linear regression function of the degree of anterior corneal astigmatism characterized a reduced positive correlation of 46.10% between the postoperative (0.6197) and preoperative values (−0.3576).

**Figure 4:**
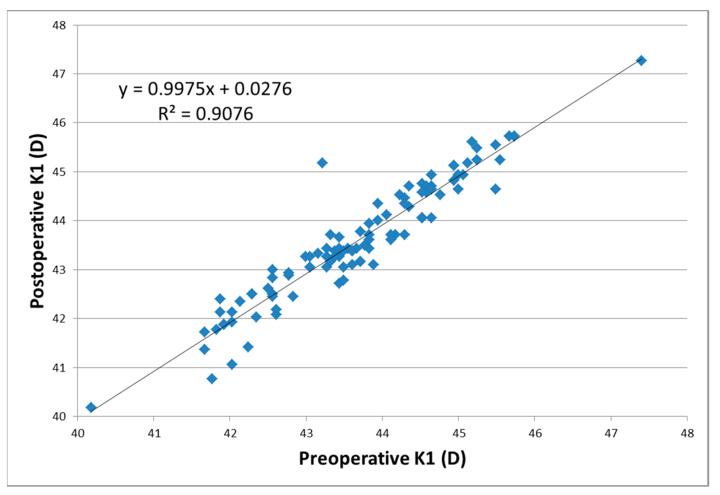
Correlation between the postoperative and preoperative values of K1.

The average K1 parameter for astigmatism (the flattest corneal point) was 43.76 ± 1.10 D (values between 41.67 and 45.67 D) in females and 43.53 ± 1.37 D in males (values between 40.18 and 47.40 D) before cataract surgery. Postoperatively, the mean K1 values were 43.69 ± 1.15 D (values within the 40.76–45.73 interval) for female patients and 43.42 ± 1.44 D (values between 40.18 and 47.27) for males. The mean pre-/post-operative differences in the K1 values was 0.08 ± 0.38 D, with a statistically significant difference between the two datasets (*p* = 0.0152). The linear regression function for K1 characterized a good positive correlation of 90.76% between the pre- and post-operative values (Figure 4).

**Figure 5:**
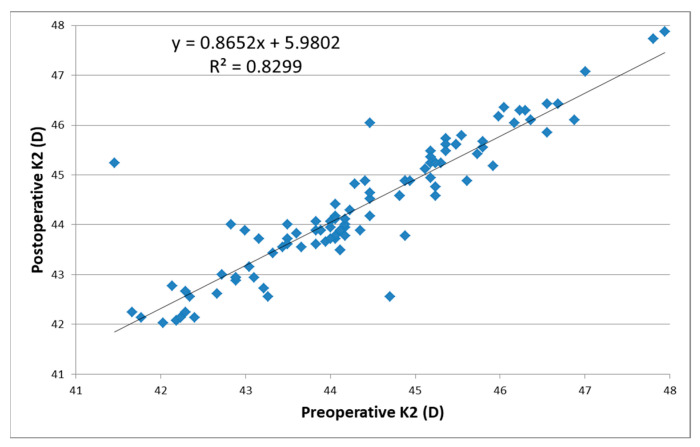
Correlation between the postoperative and preoperative values of parameter K2 for astigmatism (steepest point—steepest K).

Regarding K2 (the steepest corneal point), the preoperative averages were 44.55 ± 1.32 D (values between 41.77 and 47.94) in females and 44.09 ± 1.48 D in males (values between 41.46 and 47.80 D), while the postoperative averages were 44.51 ± 1.30 D (values within the 42.08–47.87 interval) for female patients and 44.14 ± 1.35 D (values between 42.03 and 47.74) for males. The mean pre-/postoperative differences in the K2 values was 0.002 ± 0.58 D (*p* = 0.4854). The linear regression function for K2 characterizes a moderate positive correlation (82.99%) between the pre- and post-operative values (Figure 5).

## 4. Discussion

The anterior chamber depth refers to the distance between the anterior surface of the cornea and the anterior surface of the lens, which is an indicator of the axial position of the postoperative IOL (the so-called ELP-effective lens position) and postoperative ACD prediction errors, leading to myopia or hyperopia. There are different views regarding the time needed for postoperative refractive stability, with some studies concluding that it is reached in 2 to 6 weeks after surgical intervention [22,26]. Our results highlight that the ACD deepens following cataract surgery, a statistically significant difference (*p* < 0.001) being observed between the two datasets. Similar results have been reported in recent studies, suggesting that ACD plays an important role in predicting postoperative refractive errors [22,27]. One of the causes may be that artificial IOL is thinner than the natural lens and the increase in postoperative ACD is due to an angular backward movement of the iris (of about 10°) after the lens’ removal [28].

We used a linear regression model to evaluate the correlation between pre- and postoperative ACD and pre- and postoperative LT. We found a weak positive correlation (27%) between the preoperative and postoperative ACD, and a weak negative correlation of 19.28% between the preoperative ACD and preoperative LT, both results being statistically significant. Extremely weak negative correlation (0.92% and 0.37%) was found for postoperative ACD vs. pre- and postoperative LT and correlation (0.92% and 0.37%), these results also being statistically significant.

Regarding AL, we found a statistically significant slight decrease after the surgical intervention, the results being consistent with the study of Prinz et al. on a IOLMaster device (Carl Zeiss Meditec AG, Oberkochen, Germany) [29]. Prinz et al. suggested that these postoperative AL changes might be attributed to the IOLMaster correction factors for pseudophakic AL. However, Mandal et al. concluded that there were no statistically significant differences in terms of the biometric measurements between the IOLMaster device and the Topcon Aladdin device we employed in this study [19]. Other studies have also found a statistically significant difference between pre- and postoperative AL, which may be due to differences in the characteristics of the studied population [30,31,32]. Another explanation could be that the refractive index of the lens changes due to cataract [29,33]. Biometrics always use the same refractive index for all patients, some authors having proposed an increased refractive index to minimize the AL differences [29,30,32]. Although our study included a relatively small number of patients, both preoperative ACD and AL mean values were comparable to those of other studies performed on larger samples [23,34].

An increased CCT may be observed after cataract surgery due to a number of factors: trauma and intraoperative mechanical endothelial loss, postoperative inflammation, or postoperative increases in intraocular pressure. This can temporarily affect the endothelium’s ability to maintain corneal clarity, resulting in increased corneal thickness and corneal edema [35,36,37]. Thus, the main determinant of the patient’s visual acuity on the first postoperative day is the extent to which the corneal endothelium is protected [25]. This protection is instrumental in patients prone to a greater loss of endothelial cells during phacoemulsification, influencing postoperative visual prognosis [38].

In our study, a statistically significant increase (*p* < 0.01) in corneal thickness was observed between the pre- and postoperative measurements, in accordance with the results reported by Caglar et al., Singh et al., and Behndig et al. [35,36,37]. The linear regression model revealed a reduced positive correlation of 68.89% between the pre- and postoperative CCT, the results being statistically significant. We also noticed a decrease in CCT with increased age, consistent with other studies [39]. However, the impossibility to dynamically track the batch from the preoperative period to the postoperative stabilization one was one of the limitations of this study.

Another important factor to consider in order to have a satisfactory result is the hardness of the cataract and the degree of lens opacification. The harder the lens nucleus, the more phacoemulsification energy is required, and therefore cataract density can be a predictor of the amount of energy used during phacoemulsification [40,41]. Determining the appropriate phacoemulsification strategy is very important for intraoperative and postoperative complications and for an optimal postoperative visual outcome. Analysis of the prevalence of corneal astigmatism and its characteristics may provide useful information on cataract surgery. It is well-known that the value of residual astigmatism is influenced by the location and size of the incision, the surgical procedure, the surgeon’s dexterity, and the individual biological response of each patient’s cornea [42]. Astigmatism of less than 1.00 D provides good visual acuity and patients generally do not require corrections. On the other hand, it was demonstrated that astigmatic refractive errors of 1.00 to 2.00 D significantly reduce the visual acuity [43].

The use of anterior corneal keratometry in the calculation of the residual astigmatism after cataract surgery could be criticized, as these measurements do not take into account the posterior surface of the cornea. However, Klijn et al. described changes below 0.1 D on the posterior surface, suggesting that the effect of the posterior corneal surface after cataract surgery in the calculation of residual astigmatism is of negligible clinical relevance [44].

A 2017 study by Yang et al. showed that a 2.2-mm main incision produced a relatively small residual postoperative astigmatism [45]. Their results were similar to our study as using the same incision (2.2-mm), we found a mean difference in the postoperative corneal astigmatism values of −0.09 ± 0.47 CYL, with a statistically significant (*p* = 0.0296) decrease after surgery using the 2.2-mm incision.

The main potential limitations of our study were a relatively small population from a single medical center. More studies on larger groups of patients are needed to assess the correlations between the postoperative and preoperative values of the biometric parameters. However, such results may be of interest to cataract surgeons and biometrics technicians, bringing up-to-date and supplementary information.

## 5. Conclusions

Ocular biometric measurements using OLCI technology in patients with age-related cataracts highlighted that the ACD deepened significantly postoperatively, but correlations between the pre- and postoperative ACD and pre- and postoperative LT values were weak. Regarding AL, we noticed a slight postoperative decrease after surgery, and a very good positive correlation between the post- and preoperative values. The increase in postoperative CCT observed in this study suggests that there was some disruption of the endothelial cell layer that led to a change in corneal thickness, but not to the extent that it could have caused visual impairment. Additionally, the CCT values decreased with increased age. The 2.2-mm corneal incision during cataract surgery resulted in a relatively small postoperative residual astigmatism and did not produce statistically significant changes in the refractive power of the two main meridians of the patients’ corneas.

## Figures and Tables

**Table 1:** Age distribution by gender.

Age Groups	Males	Females
Average	69.71 ± 8.35	73.44 ± 6.20
50–59 years	4	
60–69 years	13	16
70–79 years	11	29
80–90 years	3	7
Total	31 (37.3%)	52 (62.7%)

**Table 2:** Pre-/postoperative centrality and dispersion indicators of the ocular biometric and keratometric parameters.

Ocular Biometric and Keratometric Parameters	Preoperative Values Mean ± SD (min; max)	Postoperative Values Mean ± SD (min; max)	*p* Value
Axial length (mm)	23.53 ± 1.41 (21.26; 30.10)	23.46 ± 1.40 (21.45; 29.89)	<0.001
Anterior chamber depth (mm)	3.23 ± 0.51 (2.19; 5.10)	4.34 ± 0.51 (2.49; 5.18)	<0.001
Lens thickness (mm)	4.48 ± 0.35 (3.71; 5.22)	0.79 ± 0.12 (0.56; 1.00)	<0.001
Corneal central thickness (µm)	546.49 ± 35.24 (486; 634)	558.84 ± 38.23 (491; 654)	<0.01
Degree of anterior corneal astigmatism (CYL)	−0.707 ± 0.612 (−3.650; 0.970)	−0.796 ± 0.559 (−3.550; 0.000)	0.0296
The flattest point/meridian K1 (D)	43.67 ± 1.21 (40.18; 47.40)	43.59 ± 1.26 (40.18; 47.27)	0.0152
The steepest/meridian point K2 (D)	44.37 ± 1.39 (41.46; 47.94)	44.37 ± 1.32 (42.03; 47.87)	0.4854

SD—standard deviation; CYL—cylinder; D—diopters. A *p* value below 0.05 was considered statistically significant.

## Data Availability

The data presented in this study are available on request from the corresponding author.
